# Genetic variants in *LPL*, *OASL *and *TOMM40/APOE-C1-C2-C4 *genes are associated with multiple cardiovascular-related traits

**DOI:** 10.1186/1471-2350-12-123

**Published:** 2011-09-24

**Authors:** Rita PS Middelberg, Manuel AR Ferreira, Anjali K Henders, Andrew C Heath, Pamela AF Madden, Grant W Montgomery, Nicholas G Martin, John B Whitfield

**Affiliations:** 1 Genetic Epidemiology Unit, Queensland Institute of Medical Research, Brisbane, Australia; 2 Department of Medicine, Prince Charles Hospital, Queensland, Australia; 3 Department of Psychiatry, Washington University School of Medicine, St Louis MO, USA; 4 Molecular Epidemiology Unit, Queensland Institute of Medical Research, Brisbane, Australia

## Abstract

**Background:**

Genome-wide association studies (GWAS) have become a major strategy for genetic dissection of human complex diseases. Analysing multiple phenotypes jointly may improve both our ability to detect genetic variants with multiple effects and our understanding of their common features. Allelic associations for multiple biochemical traits (serum alanine aminotransferase, aspartate aminotransferase, butrylycholinesterase (BCHE), C-reactive protein (CRP), ferritin, gamma glutamyltransferase (GGT), glucose, high-density lipoprotein cholesterol (HDL), insulin, low-density lipoprotein cholesterol (LDL), triglycerides and uric acid), and body-mass index, were examined.

**Methods:**

We aimed to identify common genetic variants affecting more than one of these traits using genome-wide association analysis in 2548 adolescents and 9145 adults from 4986 Australian twin families. Multivariate and univariate associations were performed.

**Results:**

Multivariate analyses identified eight loci, and univariate association analyses confirmed two loci influencing more than one trait at p < 5 × 10^-8^. These are located on chromosome 8 (*LPL *gene affecting HDL and triglycerides) and chromosome 19 (*TOMM40/APOE-C1-C2-C4 *gene cluster affecting LDL and CRP). A locus on chromosome 12 (*OASL *gene) showed effects on GGT, LDL and CRP. The loci on chromosomes 12 and 19 unexpectedly affected LDL cholesterol and CRP in opposite directions.

**Conclusions:**

We identified three possible loci that may affect multiple traits and validated 17 previously-reported loci. Our study demonstrated the usefulness of examining multiple phenotypes jointly and highlights an anomalous effect on CRP, which is increasingly recognised as a marker of cardiovascular risk as well as of inflammation.

## Background

Genome-wide association studies (GWAS) have become a major strategy for genetic dissection of human complex diseases. There is substantial overlap, both phenotypically and in allelic associations, between biomarkers and/or risk factors and between related diseases, and it is becoming important to understand the ways in which polymorphisms affect multiple phenotypes. Many phenotypes may be available from a single study population but current GWAS approaches usually examine them separately within a univariate framework. This strategy ignores potential genetic correlation between different traits.

From the perspective of maximising power for a given size of dataset, it has been shown that joint analyses of correlated traits in linkage analysis have substantially improved power in localizing genes [1-4]. Similarly, multivariate approaches in association studies can theoretically improve the ability to detect genetic variants whose effects are too small to be detected in univariate tests [4]. Multivariate association tests have been proposed for unrelated samples [5] and for family data [6]. Most of these tend to be inefficient and/or computationally intensive, especially at the genome-wide level. The approach proposed by Ferreira and Purcell has been shown to be powerful when traits have moderate to high correlation and efficient when applied to samples of unrelated individuals [7].

Genetically complex (multifactorial) diseases such as cardiovascular disease and type 2 diabetes often have common risk factors. A number of biochemical markers are known to be associated with obesity, pre-diabetic states, or risk of cardiovascular disease. Lipid traits such as triglycerides, and the low-density lipoprotein (LDL) and high-density lipoprotein (HDL) components of cholesterol, are well-known risk factors for cardiovascular disease. Other biochemical markers such as C-reactive protein (CRP) [8], the enzymes used as liver function tests (gammaglutamyl transferase, GGT [9-11], alanine aminotransferase, ALT; and aspartate aminotransferase, AST), butyrylycholinesterase (BCHE) [12,13]), serum ferritin [14] and uric acid [15,16] have also been shown to be associated with the risk of cardiovascular disease, hypertension, obesity, insulin resistance or metabolic syndrome. These biochemical markers are correlated so we may gain power, insight or both from a multivariate approach. For example, serum GGT is significantly correlated with total or LDL cholesterol, HDL (inversely) and particularly with triglycerides [17,18]. Also, GGT is significantly correlated with other liver enzymes AST and ALT [17,19]. Serum triglyceride is correlated with the liver enzymes [17] and uric acid and also associated with cardiovascular risk.

The importance of genetic variation has been shown previously through univariate analyses of serum lipids [20], uric acid [21-23], GGT [24], ALT [24] and AST [17,24], BCHE [25], ferritin [26] and for CRP [27,28]. Nevertheless, little is known about common genetic influences on these variables and joint analysis may reveal whether the same gene influences multiple traits.

The aim of our study is to identify genes and regions associated with multiple biochemical traits related to cardiovascular risk, type 2 diabetes or metabolic syndrome. We used a recently described multivariate association test [7] to perform genome-wide association analysis. This approach was used initially to screen for multivariate trait-SNP association using a subset of unrelated individuals. To confirm findings from the multivariate test, univariate association tests were conducted making use of the full dataset by including all family members.

## Methods

### Subjects

Biochemical traits were measured in serum samples from twins and their families, and genome-wide SNP markers were genotyped. The study participants comprise:

(1) Adolescent twins and their non-twin siblings living in south-east Queensland (Australia) who had participated in the Brisbane Longitudinal Twin study [29-32]. Full details are described in Middelberg et al. [33]. A total of 2548 participants (1317 females and 1231 males; mean age of 14.8 years) were genotyped.

(2) Adult twins consisting of twins and their family members who participated in studies of: (i) alcohol and nicotine dependence and metabolic risk for alcoholic liver disease [34]; (ii) anxiety and depression [35]; and (iii) endometriosis [36]. A total of 9145 individuals (5703 females and 3442 males; mean age of 46.2 years) were genotyped.

Combining these studies, 20,230 individuals had biochemical measurements and 11,683 (from 4986 families) had both genotype and phenotype data. Out of the 11,683, there are only 1483 (from 1015 families) who had data for all the 13 traits. Where multiple measurements of the same trait in an individual were available, the average of the values was used.

For each of these studies, participants (and, for subjects aged < 18 years, their parents) gave informed consent to the questionnaire, interview, and blood collection, and all studies were approved by the QIMR Human Research Ethics Committee.

### Laboratory measurements

Serum was separated from the blood samples and stored at -70°C until analyzed. Serum cholesterol, HDL cholesterol, triglycerides, BCHE, glucose, uric acid, ferritin, CRP, AST, ALT and GGT were measured using Roche methods on a Roche 917 or Modular P analyzer (Roche Diagnostics, Basel, Switzerland). LDL cholesterol was calculated using the Friedewald equation. Insulin was measured on an Abbott Architect. BMI was calculated from measured or self-reported weight and height for the adults and from measured weight and height for the adolescents.

### Genotyping

DNA was extracted from blood samples using standard methods and genotyped with Illumina 610K, 317K or 370K chips at CIDR or deCODE Genetics. Data cleaning for SNP genotypes included checking the expected relationships between individual family members and resolving Mendelian errors [37]. Imputed genotypes for non-typed HapMap SNPs were generated using *MACH1.0 *(http://www.sph.umich.edu/csg/abecasis/mach/index.html) [38-40] program. Any imputed SNP which had *r*^2 ^≥ 0.3 was included in the genotype data.

### Statistical Analysis

Distributions of all biochemical variables were examined. Serum AST, ALT and GGT, CRP, triglycerides and BMI were log-transformed. For each trait, individuals who were more than five standard deviations from the mean of that trait were excluded. Results for glucose and insulin in adults were adjusted for fasting time based on the reported time of last meal and time of blood collection. Prior to genetic association analysis, the variables were also adjusted for the effects of age, squared age (age^2^), sex, sex × age and sex × age^2^. Standardized residuals were obtained and used in the association analysis. All data pre-processing and descriptive analyses were done using *STATA *version 7.0 [41] and *SPSS *version 17.0.2 (Mar 11, 2009). Multivariate association analysis was performed using the *PLINK *(v1.07) implementation of the multivariate test described by Ferreira and Purcell [7]. This test is computationally too intensive when applied to family data to be efficient for genome-wide analysis. Therefore the analysis was performed in two stages. First, we selected one individual per family (using the person with data for the greatest number of phenotypes) from each of the 4986 families and applied the multivariate test as a screening tool. Next, for each locus with a multivariate p-value of less than 5 × 10^-8^, we identified the traits that showed evidence for association with that locus (that is, with a canonical correlation weight > |0.2|) and confirmed that specific trait-SNP association with a univariate association test using all relatives for each family. The univariate association test was performed using "*fastassoc*" in *MERLIN 1.1.2 *[42] which takes the average of two results in MZ twin pairs.

## Results

### General Characteristics

Means and standard deviations of all the traits for males and females in adolescent and adult genotyped cohorts are listed in Additional file 1, Table S1. Generally, the means of the biochemical traits are lower in the adolescents than the adults, as expected. Phenotypic correlations between each pair of age-corrected traits separately for males and females in the combined sample are shown in Additional file 1, Table S2. The strongest correlations (r > 0.5) observed in males were between glucose and insulin (0.53), between AST and ALT (0.66) and between GGT and ALT (0.57). In the females, the strongest correlations observed were between glucose and insulin (0.59), between BCHE and glucose (-0.59), between BCHE and CRP (-0.53) and between AST and ALT (0.63). Given that most of the other pair-wise correlations (Additional file 1, Table S2) are low to moderate (r < 0.3), the multivariate approach is expected to provide comparable or slightly improved power to detect pleiotropic loci when compared to univariate analysis followed by correction for the number of traits tested [7].

### Genome-wide association analyses

**Figure 1:**
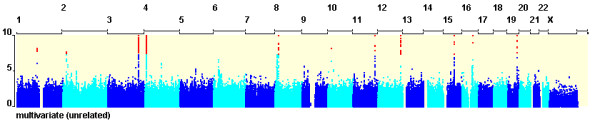
**Manhattan plots for multivariate QTL analysis in unrelated-subject data (N = 4986) for the 13 traits**. Genomic position is on the x-axis and the -log_10 _of the association p-value is on the y-axis. Points with p-value of 5 × 10^-8 ^are shown in red.

The multivariate analysis identified a total of 766 SNPs in 11 independent (r^2 ^< 0.1) loci associated with biochemical traits with a p-value of less than 5 × 10^-8 ^(Table 1 and Figure 1). Of these, there are eight loci potentially associated with more than one trait (Table 1). Three loci (on chromosomes 8, 12 and 19) showed strong or close to genome-wide significant evidence of associations with more than one trait in the all-subject univariate analyses.

**Table 1:** Summary of SNP associations (based on multivariate p-value of < 5 × 10^-8^)

Chr	BP	SNP	Closest Gene	Minor/Major Allele	MAF	N	multivariate P-value	Associated Trait(s)	Univariate analysis			
									
									N	β*	SE	univariate p
1	109,619,361	rs660240	*CELSR2*	T/C	0.215	4883	7.0 × 10^-9^	**LDL**	**11247**	**-0.170**	**0.017**	**2.1 × 10**^**-22**^
								HDL	11545	0.047	0.017	0.007
								INS	2559	0.035	0.040	0.390
2	21,097,505	rs10199768	*APOB*	T/G	0.470	4872	2.3 × 10^-8^	**LDL**	**11205**	**0.110**	**0.014**	**7.7 × 10**^**-15**^
								CRP	8878	-0.015	0.015	0.330
								FERR	11285	0.032	0.014	0.020
3	166,973,974	rs1803274	*BCHE*	T/C	0.206	4883	2.4 × 10^-42^	**BCHE**	**9171**	**-0.365**	**0.018**	**5.9 × 10**^**-92**^
4	9,665,474	rs7671266	*WDR1*	T/C	0.208	4883	1.8 × 10^-42^	**UA**	**11346**	**-0.305**	**0.017**	**9.3 × 10**^**-71**^
4	9,940,392	rs4698036	Intergenic	G/T	0.218	4884	8.5 × 10^-31^	**UA**	**11354**	**-0.257**	**0.017**	**2.3 × 10**^**-52**^
**8**	19,894,037	**rs17091905**	***LPL***	A/G	0.124	4884	2.8 × 10^-13^	**HDL**	**11546**	**0.150**	**0.022**	**5.7 × 10**^**-12**^
								**TRIG**	**11576**	**-0.174**	**0.022**	**5.1 × 10**^**-15**^
								CRP	8918	0.062	0.024	0.008
								BCHE	9168	-0.042	0.032	0.069
10	17,931,828	rs2437258	*MRC1*	T/C	0.226	4884	7.1 × 10^-9^	**AST**	**11516**	**0.102**	**0.016**	**3.0 × 10**^**-10**^
								FERR	11332	0.015	0.016	0.350
								TRIG	11579	0.027	0.017	0.116
								ALT	11518	-0.012	0.017	0.460
**12**	119,955,720	**rs3213545**	***OASL***	A/G	0.295	4876	3.9 × 10^-14^	**GGT**	**11493**	**-0.121**	**0.015**	**3.6 × 10**^**-15**^
								LDL	11228	0.066	0.016	2.9 × 10^-5^
								CRP	8903	-0.066	0.017	8.8 × 10^-5^
								TRIG	11556	0.021	0.016	0.180
15	56,465,804	rs10468017	*LIPC*	T/C	0.326	4864	2.8 × 10^-11^	**HDL**	**11497**	**0.104**	**0.015**	**2.8 × 10**^**-12**^
								TRIG	11527	0.048	0.015	0.002
16	55,545,545	rs173539	*CETP*	T/C	0.324	4884	1.7 × 10^-40^	**HDL**	**11549**	**0.255**	**0.015**	**3.3 × 10**^**-65**^
								LDL	11251	-0.050	0.015	0.001
**19**	50,087,459	**rs2075650**	***TOMM40***	G/A	0.152	4884	5.7 × 10^-10^	**LDL**	**11248**	**0.153**	**0.020**	**1.6 × 10**^**-14**^
								**CRP**	8918	-0.116	0.021	**4.2 × 10**^**-8**^
								HDL	11546	-0.105	0.020	8.1 × 10^-8^
								TRIG	11576	0.098	0.020	9.6 × 10^-7^

SNPs highlighted in bold indicate the polymorphisms that are significantly associated with more than one trait; traits highlighted in bold are those which achieved significance in the univariate analysis.

* β represents 1 unit change in e.g. LDL (*mmol/l*) per copy increment in the minor allele; TRIG indicates triglycerides.

The most strongly associated SNP at the chromosome 8p21.3 locus was rs17091905 (multivariate p = 2.8 × 10^-13^). HDL, CRP, triglycerides and BCHE had trait loadings of greater than |0.2|. To confirm the multivariate result, we individually tested each of these traits using a univariate test in the full sample of 11,683 individuals. The univariate tests confirmed the association with HDL (p = 5.7 × 10^-12^) and triglycerides (p = 5.1 × 10^-15^) but not at genome-wide significance for CRP (p = 0.008) and non-significant (p = 0.069) for BCHE. This variant is in strong or partial LD with previously-reported variants for HDL or triglycerides [43-45] (Additional file 1, Table S3).

The second variant rs3213545 (multivariate p-value = 3.9 × 10^-14^) which is located on chromosome 12q24.2 (*OASL*) was confirmed to be significantly associated with GGT (p = 3.6 × 10^-15^) [46] and also showed moderately strong significance for LDL (p = 2.9 × 10^-5^) and CRP (p = 8.8 × 10^-5^) (Table 1).

**Figure 2:**
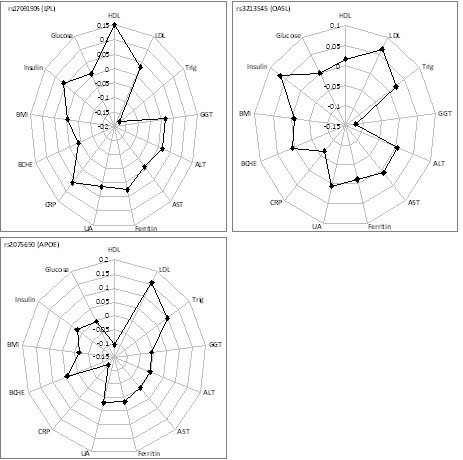
**Radar chart of polymorphisms on chromosome8 (a), chromosome 12 (b) and chromosome 19 (c)**. Each dot on the plot represents the standardized beta (1-unit change per copy increment of the minor allele) of each trait from univariate testing.

The third variant was rs2075650 (multivariate p-value = 5.7 × 10^-10^) located on chromosome 19q13.32 (*TOMM40/APOE-C1-C2-C4 *gene cluster) where HDL, LDL, CRP and triglycerides had trait loadings of greater than |0.2|. Significant univariate associations were observed for LDL (p = 1.6 × 10^-14^) and CRP (p = 4.2 × 10^-8^) and close to genome-wide significant univariate associations were seen for HDL (p = 8.1 × 10^-8^) and triglycerides (p = 9.6 × 10^-7^) (Table 1 and Figure 2). This SNP has previously been reported to be associated with LDL [43], LDL buoyancy [47] and CRP [48], and there is an association between LDL (or TG or HDL) and rs4420638 which is in partial LD (r^2 ^= 0.4) with this SNP (Additional file 1, Table S3).

To determine whether there are any further unreported genes/regions to be detected by multivariate analysis, a lower p-value threshold of multivariate p < 9 × 10^-5 ^was used. No new loci were found but a further six previously reported loci were replicated as listed in Additional file 1, Table S4.

**Figure 3:**
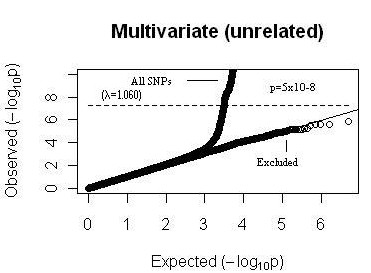
**Q-Q plot of multivariate analysis**. Black points correspond to SNPs included in the analyses. The 45° line refers to no significant association. The dotted line corresponds to p-value of 5 × 10^-8^. "Excluded" line is where SNPs that were found in significant regions (genes) in univariate analyses were removed.

The Q-Q plot from multivariate analysis was also examined closely to determine whether there are any excess association signals detected by multivariate analysis which have not already been detected by univariate analysis. SNPs that were found in significant regions (genes) in univariate analyses were removed (Figure 3) from the plot. The Q-Q plot with excluded SNPs showed that there is no excess of significant p-values hence indicating there are additional loci that have not already been detected by univariate analysis.

Examination of the directions of the allelic effects on the different phenotypes showed unexpected results. At LPL on chromosome 8, the minor allele A at rs17091905 increased HDL-C and decreased triglycerides, but the direction of the nominally significant effect on CRP was to increase it. At the chromosome 12 locus the minor allele A at rs3213545 tended to increase LDL-C but it significantly decreased GGT and tended to decrease CRP. Similarly at the chromosome 19 locus, the effect of the minor allele (G for rs2075650) was to increase LDL-C and triglycerides and to decrease HDL-C, consistent with an increase in cardiovascular risk, but to decrease CRP, again suggesting opposite allelic effects on the markers of different aspects of cardiovascular risk.

## Discussion

We have applied a multivariate approach to identify variants associated with more than one trait, initially using 4986 unrelated individuals across 13 biochemical traits. Univariate testing of the significant or near-significant loci, on the full sample of 11,683 individuals, was then used to confirm these findings. We are interested firstly in the usefulness of multivariate analysis as a substitute for the more laborious and potentially less powerful approach of conducting multiple univariate analyses and comparing the results, and secondly in the details of the loci which are found to have effects on multiple variables in our data.

Testing one individual per family identified three known loci that were significantly or near-significantly associated with more than one trait, and replicated 11 loci in previously published genes that that passed a genome-wide threshold of 5 × 10^-8 ^for single variables (Table 1). When a lower genome-wide threshold (p < 9 × 10^-5^) was used, a further six published loci were also identified (Additional file 1, Table S4). The three loci in previous publications using univariate association analysis (highlighted in Table 1) had evidence of significant or close to significant associations with more than one trait in our data, hence indicating benefits of detecting pleiotropic loci in multivariate analysis.

We have identified polymorphisms showing strong evidence of allelic associations with HDL and triglycerides on chromosome 8 (*LPL *gene MIM 609708); with GGT and possibly LDL and CRP on chromosome 12 (*OASL *gene MIM 603281); and with HDL and LDL and possibly CRP and triglycerides on chromosome 19 (*TOMM40 *(MIM 608061) */APOE *(MIM 107741)*-C1 *(MIM 107710)*-C2 *(MIM 608083)*-C4 *(MIM 600745) gene cluster). Each gene has been previously recognised in genome-wide association studies concentrating on a few of these variables [49]. The function of these genes is reasonably well-established. *LPL *plays a key role in lipid metabolism and is responsible for hydrolysis of triglyceride molecules present in circulating lipoprotein. *APOE *and *APOC *genes also play a key role in lipid metabolism and cholesterol transport by helping to stabilise and solubilize lipoproteins as they circulate in the blood [50,51]. Both *LPL *and *APOE *polymorphisms have been found to be significantly associated with increases in LDL and decreases in HDL [52]. The functional connection between the *OASL *gene (2',5'-oligoadenylate synthetase-like, also known as "thyroid hormone receptor interactor") and these phenotypes is unclear. However, nearby genes in linkage disequilibrium with the lead SNP in *OASL *include *HNF1A *and *c12orf43*. *HNF1A *is expressed in liver, kidney and endocrine pancreas and regulates a number of genes involved in lipoprotein metabolism including apolipoproteins, cholesterol synthesis enzymes and bile acid transporters [53]. *HNF1A *also has allelic associations with type 2 diabetes [54], CRP [55-57] and coronary heart disease [58]. The findings for the lipids, in particular, were similar to those obtained in previous genetic association studies on general population. However the relationships between inflammation (as presumptively measured by CRP) and the traits associated with obesity and cardiovascular risk are of particular interest. CRP was significantly, though not always strongly, correlated with each of the other traits at the phenotypic level and it also showed up in the multivariate association findings.

The multivariate approach helps us to understand the connections between variables. For example, for rs2075650 on chromosome 19, the multivariate approach suggested LDL, CRP, HDL and triglycerides might be associated with this particular SNP. Although the effects on LDL, HDL and triglycerides are consistent with what was expected (that is, the LDL effect is inversely associated with the HDL effect, positively associated with the triglycerides effect, and the HDL effect is inversely associated with that on triglycerides), the effects on CRP are contrary to expectation. The effect direction is opposite to those for LDL and triglycerides, and the same as that for HDL. This suggests that the alleles or haplotype which have risk-increasing effects on lipids have a potentially protective effect on CRP and (so far as the effect on CRP is reflecting the degree of inflammation) on the inflammatory process. The effect estimates of LDL for rs2075650 obtained in our study were similar to obtained by Aulchenko et al. [43]. The effect of rs2075650 [G] on LDL was estimated as 0.160 ± 0.018 by Aulchenko et al. and 0.153 ± 0.020 in our analysis. The effect estimates of CRP for this SNP was not available from previous study for comparison. In addition, it was interesting to observe that rs4429638 which is in partial LD (r^2 ^= 0.4) with rs2075650 has allelic effects in the opposite direction on LDL [52,59-61] and CRP [55]. Similarly on chromosome 12, rs3213545 affects LDL and CRP (and GGT) but not HDL or triglycerides. Again, the allelic effects on LDL and CRP are in opposite directions. This shows the usefulness of the multivariate approach to help understand the connections between several trait-SNP associations, which can then be modelled and evaluated in more detail.

Our study differs from previous investigations as it examines a large number of correlated biochemical traits, initially using unrelated individuals and following up the findings in other members of the families. It confirms some published associations and identifies new ones. As our cohort consisted of adolescents and adults, results from adults, adolescents and combined (adults and adolescents) cohorts were examined and compared. Because of the larger number of adults studied, results from adults were similar to the combined data. Results from the adolescents were not notably different from the combined data.

One main limitation of our approach is that only a subset of the data (from unrelated subjects) can be used for the initial multivariate analysis. Although it would add power, it is too computationally intensive to use all the available data (that is, taking account of the family structure) in genome-wide multivariate analysis. Although a subset of data was used, the method applied in our study was very efficient and easy to perform. A more specific limitation in our data is that the glucose and insulin measurements were not made on fasting blood samples. In adults, we made adjustments for the time since the last meal but in adolescents we had to rely on the fact they were seen at the same time of day and blood was taken around three hours after the expected time of breakfast.

Another set of limitations is related to the use of biomarkers of risk or, for CRP, of systemic inflammation. It seems that some loci may affect HDL-C or triglycerides without affecting cardiovascular risk [49], and it is possible that some loci might affect serum CRP without affecting inflammation. Nevertheless the divergence between allelic effects on risk factors deserves further examination.

## Conclusion

Our study demonstrated that it is useful to examine multiple phenotypes jointly in order to better understand the connections between them and to make the distinction between common and unique genetic effects. Our efficient approach (a combination of multivariate and univariate analysis) was able to identify three possible loci that might affect multiple traits, and validated 17 loci that have previously been reported. It highlighted anomalous effects on CRP, which is increasingly recognised as a marker of cardiovascular risk as well as of inflammation. Confirmation and extension of our findings will require studies which measure multiple phenotypes in each genotyped subject, and will benefit from combination of data from multiple studies to achieve sufficient power.

## Competing interests

The authors declare that they have no competing interests.

## Authors' contributions

RPSM analysed data, interpreted and prepared the manuscript. MARF and JBW contributed to data interpretation and manuscript preparation. AKH and GWM coordinated genotyping studies. ACH, PAFM and NGM coordinated the phenotype studies. All authors read and approved this manuscript.

## Pre-publication history

The pre-publication history for this paper can be accessed here:

http://www.biomedcentral.com/1471-2350/12/123/prepub

## Supplementary Material

Additional file 1 **Additional tables and references**. Additional Tables S1-S4 and references cited in these tables. Table S1 gives a descriptive statistics for males and females in the two genotyped cohorts. Table S2 gives the phenotypic correlations between each pair of age-corrected traits separately for males and females in combined sample. Table S3 compares the multivariate and univariates results from our study with published results. Table S4 gives a summary of borderline significant associations from multivariate analysis. Click here for file
