# Supplementary material for: Genetic variants in LPL, OASL and TOMM40/APOE-C1-C2-C4 genes are associated with multiple cardiovascular-related traits
Source: BMC Med Genet. 2011 Sep 24;12:123. doi: 10.1186/1471-2350-12-123 (PMC3189113; doi:10.1186/1471-2350-12-123)
Supplement: Additional file 1 — Additional tables and references. Additional Tables S1-S4 and references cited in these tables. Table S1 gives a descriptive statistics for males and females in the two genotyped cohorts. Table S2 gives the phenotypic correlations between each pair of age-corrected traits separately for males and females in combined sample. Table S3 compares the multivariate and univariates results from our study with published results. Table S4 gives a summary of borderline significant associations from multivariate analysis. [file 1471-2350-12-123-S1.PDF]

Additional tables and references

for "Genetic variants in *LPL, OASL* and *TOMM40/APOE-C1-C2-C4* genes are associated with multiple cardiovascular-related traits "

Contents

Table S1. Descriptive statistics for adult and adolescent genotyped cohorts....……..……......2

Table S2. Phenotypic correlations of age-corrected traits by sex. Results for women are shown below the diagonals and for men above. Numbers on which correlations are based are shown in parenthesis; numbers on the diagonals are total numbers for females and males. Symbol ∅ indicated p-value of correlation is > 0.05……………..………………...…...........4

Table S3. Comparisons of multivariate (p-value <5 x10^{-8}) and univariate results from our study with published results. Highlighted in bold indicates significant associations (multivariate p-value <5 x10^{-8}). Highlighted in bold and italics indicates significant associations (multivariate p-value <5 x10^{-8}) with a new trait……….…………..…………7

Table S4. Summary of borderline significant associations (multivariate p-value of < 9x10^{-5} and > 5x10^{-8}).………………………………………………………...…………....…...21

Additional References ………….……………………...……..……….…………. 23

Table S1: Descriptive statistics for adult and adolescent genotyped cohorts

**Trait**	**Adult**						**Adolescent**					
	Males			Females			Males			Females		
	N	Mean	SD	N	Mean	SD	N	Mean	SD	N	Mean	SD
**Age (*years*)**	3434	47.08	12.46	5688	45.70	12.90	1231	14.64	1.86	1317	15.00	2.26
**HDL (*mmol/l*)**	3381	1.33	0.35	5639	1.64	0.41	1228	1.34	0.29	1317	1.44	0.29
**LDL (*mmol/l*)**	3181	3.43	0.91	5545	3.21	0.93	1224	2.39	0.67	1317	2.48	0.64
**TRIG(*mmol/l*)**	3389	0.28	0.24	5661	0.15	0.22	1228	0.05	0.19	1317	0.02	0.17
**BMI (kg/m^2)**	2038	26.68	4.11	2867	25.45	4.99	1207	20.55	3.61	1293	20.79	3.58
**Insulin$^{\$}$($log_{10}$)(p*mol/l*)**	1003	1.66$^{\$}$	0.48	1543	1.43$^{\$}$	0.46	–	–	–	–	–	–
**Glucose$^{\$}$(*mmol/l*)**	2657	5.23$^{\$}$	1.66	3550	4.91$^{\$}$	1.33	983	3.75	1.18	1052	3.55	1.11
**CRP (log_{10}) (*mg/L*)**	3200	0.34	0.61	4970	0.60	0.92	384	–2.31	0.46	383	–2.22	0.51
**Uric Acid ($\mu mol/l$)**	3373	0.35	0.07	5450	0.26	0.07	1229	0.30	0.07	1317	0.25	0.05
**ALT(log_{10}) (*units/l*)**	3377	1.44	0.21	5595	1.24	0.20	1229	1.19	0.18	1317	1.12	0.16
**AST (log_{10}) (*units/l*)**	3375	1.41	0.14	5595	1.32	0.13	1229	1.38	0.10	1317	1.31	0.10
**GGT (log_{10}) (*units/l*)**	3377	1.46	0.29	5593	1.23	0.27	1229	1.13	0.14	1317	1.05	0.14

**Ferritin (log_{10}) (*mg/l*)**	3284	2.29	0.34	5518	1.81	0.40	1228	1.70	0.23	1317	1.55	0.27
**BCHE (*units/l*)**	3222	9286.2	2719.9	5313	7116.5	3576.5	331	8945.8	1501.0	325	8318.0	1512.5

$: adjusted for time between last mail and blood collection (fasting time) in association analysis; CRP:C-reactive protein; TRIG: Triglycerides; UA:Uric acid: AST: aspartate aminotransferase; GGT: Gamma glutamyltransferase; BCHE: Butrylycholinesterase.

Table S2: Phenotypic correlations of age-corrected traits by sex. Results for women are shown below the diagonals and for men above. Numbers on which correlations are based are shown in parenthesis; numbers on the diagonals are total numbers for females and males. Symbol ∅ indicated p-value of correlation is > 0.05

	HDL	LDL	TRIG	BMI	Ins	Gluc	CRP	UA	ALT	AST	GGT	Ferritin	BCHE
**HDL**	**(12416, 7856)**	–0.05 (7472)	–0.44 (7854)	–0.26 (4658)	–0.43 (1703)	–0.12 (5032)	–0.17 (6240)	–0.20 (7719)	–0.11 (7809)	0.06 (7807)	–0.02∅ (7866)	–0.06 (7692)	–0.01∅ (6340)
**LDL**	–0.14 (12211)	**(12211, 7472)**	0.18 (7471)	0.18 (4486)	0.00∅ (1599)	–0.10 (4833)	0.12 (5883)	0.09 (7347)	0.19 (7434)	0.06 (7432)	0.17 (7434)	0.22 (7311)	0.06 (5974)
**TRIG**	–0.40 (12413)	0.19 (12208)	**(12460, 7893)**	0.36 (4696)	0.15 (1726)	–0.06 (5055)	0.07 (6257)	0.21 (7754)	0.36 (7847)	0.19 (7845)	0.34 (7847)	0.23 (7724)	0.22 (6361)
**BMI**	–0.27 (5665)	0.16 (5608)	0.27 (5709)	**(5715, 4702)**	0.09 (1255)	–0.14 (3663)	0.19 (3150)	0.32 (4566)	0.44 (4654)	0.12 (4652)	0.36 (4654)	0.29 (4552)	0.16 (3224)
**Ins**	–0.29 (2331)	0.12 (2288)	0.10 (2353)	0.03∅ (1760)	**(2355, 1729)**	0.53 (1542)	0.40 (1466)	0.20 (1725)	–0.05 (1727)	–0.17 (1727)	–0.08 (1727)	–0.05 (1712)	–0.33 (1549)

**Gluc**	–0.16	0.04	0.03	–0.09	0.59	**(6372,**	0.34	0.06	–0.27	–0.17	–0.12	–0.16	–0.57
	(6348)	(6278)	(6368)	(4204)	(2192)	**5057**)	(3764)	(5057)	(5057)	(5057)	(5057)	(5042)	(3821)
**CRP**	-0.10	0.13	0.07	0.12	0.48	0.46	**(10289,**	0.18	0.03	–0.07	0.15	0.14	–0.48
	(10219)	(10031)	(10238)	(3659)	(1862)	(4797)	**6312)**	(6197)	(6242)	(6242)	(6243)	(6253)	(6132)
**UA**	–0.23	0.07	0.19	0.25	0.18	0.21	0.11	**(12047,**	0.20	0.10	0.22	0.16	–0.03
	(12004)	(11809)	(12044)	(5308)	(2351)	(6369)	(10050)	**7757)**	(7756)	(7754)	(7757)	(7591)	(6278)
**ALT**	-0.09	0.09	0.22	0.25	–0.11	–0.18	–0.05	0.11	(**12320,**	0.66	0.57	0.37	0.28
	(12272)	(12072)	(12316)	(5568)	(2352)	(6371)	(10180)	(12046)	**7849)**	(7847)	(7849)	(7680)	(6338)
**AST**	0.04	0.04	0.12	–0.15	–0.14	–0.09	-0.08	0.09	0.63	(**12319,**	0.38	0.18	0.17
	(12272)	(12072)	(12315)	(5567)	(2353)	(6372)	(10180)	(12046)	(12318)	**7847**)	(7847)	(7680)	(6338)
**GGT**	–0.07	0.08	0.24	0.24	–0.11	–0.10	0.08	0.17	0.45	0.27	**(12317,**	0.33	0.19
	(12271)	(12071)	(12314)	(5568)	(2352)	(6370)	(10178)	(12045)	(12316)	(12316)	**7850)**	(7681)	(6339)
**Ferritin**	–0.008	0.06	0.11	0.07	–0.08	–0.02∅	0.03	0.16	0.17	0.08	0.21	**(12232,**	0.07
	(12187)	(11983)	(12224)	(5530)	(2324)	(6339)	(10225)	(11822)	(12087)	(12087)	(12085)	**7727)**	(6356)
**BCHE**	–0.02∅	0.005∅	0.21	0.16	–0.47	–0.59	–0.53	0.03	0.27	0.14	0.20	0.08	**(10688,**

(10598)	(10403)	(10631)	(3961)	(2049)	(4959)	(10083)	(10336)	(105337)	(10537)	(10535)	(10611)	**6364)**

Table S3: Comparisons of multivariate (p-value <5 x10^{-8}) and univariate results from our study with published results. Highlighted in bold indicates significant associations (multivariate p-value <5 x10^{-8}). Highlighted in bold and italics indicates significant associations (multivariate p-value <5 x10^{-8}) with a new trait

**CHR**	**BP**	**SNP**	**Multivariate p-value (unrelated)**	**Traits with loadings >|0.2|**	**Univariate p-value (all individuals)**	**Trait (in published association)**	**Ref**	**Reported Gene(s)**
1	109618715	rs7528419	1.65X10^{-8}	HDL, LDL, INS, GLUC	0.006, **4.1x10^{-21}**, 0.40, 0.62	LDL, APOB	[4]	*CELSR2*
1	109619113	rs12740374	8.70X10^{-9}	HDL,LDL	0.006, **8.4X10^{-22}**	LDL	[5]	*CELSR2*
						APOB	[4]	*CELSR2*
1	109619829	rs629301	9.37 X10^{-9}	HDL,LDL	0.007, **5.7X10^{-22}**	LDL, APOB	[4, 6]	*SORT1*
						TC	[6]	*SORT1*
1	109620053	rs646776	9.37X10^{-9}	HDL,LDL	0.007, **5.7X10^{-22}**	TC	[7]	*CELSR2*

						Myocardial infarction (early onset)	8	*CELSR2*, *PSRC1*, *SORT1*
						LDL	5, 9	*CELSR2*, *PSRC1*, *SORT1*
						APOB	4	*CELSR2*
1	109623034	rs602633	$1.00\ X10^{-8}$	HDL, LDL, INS	0.024. $\mathbf{1.2x10^{-22}}$, 0.26	LDL, APOB	4	*CELSR2*
1	109623689	rs599839	$1.12X10^{-8}$	HDL,LDL, GLUC	0.024, $\mathbf{4.6X10^{-22}}$**,** 0.52	Coronary disease	10	*PSRC1*
						TC	11	*CELSR2*
						APOB	4	*CELSR2*
						LDL	12, 13	*CELSR2*, *PSRC1*

2	21085700	rs693	$3.06X10^{-8}$	LDL,FERR, TG	**$1.8X10^{-15}$**, 0.021, 0.022	LDL	[7]	*APOB*
						TC	[7]	*APOB*
						TG	[5, 14]	*APOB*
						LDL	[9]	*APOB*
2	21097505	rs10199768	$2.28X10^{-8}$	LDL,CRP, FERR	**$7.7 X10^{-15,}$** 0.33, 0.020	LDL	[4]	*APOB*
3	166973974	rs1803274	$2.43X10^{-42}$	BCHE	**$5.20X10^{-92}$**	BCHE	[15]	*BCHE*
4	9524839	rs11722228	$3.01X10^{-9}$	UAC	**$2.9X10^{-35}$**	UAC	[2, 16]	*SLC2A9*
4	9531265	rs16890979	$6.24X10^{-38}$	UAC	**$3.6X10^{-66}$**	UAC	[2, 17, 18]	*GLUT9, WDR1*
4	9532102	rs734553	$4.19X10^{-40}$	UAC	**$1.2X10^{-75}$**	UAC	[2]	*SLC2A9*
4	9536065	rs13129697	$1.04X10^{-37}$	UAC	**$4.3X10^{-68}$**	UAC	[2, 19]	*SLC2A9*
4	9543842	rs737267	$2.41X10^{-40}$	UAC	**$4.5X10^{-72}$**	UAC	[2, 20]	*SLC2A9*
4	9545008	rs6855911	$2.41X10^{-40}$	UAC	**$4.5X10^{-72}$**	UAC	[2, 21]	*GLUT9*

4	9575478	rs7442295	5.98X10^{-41}	UAC	**2.9X10^{-76}**	UAC	2, 13, 22	*SLC2A9, WDR1*
4	9933258	rs9291683	2.64X10^{-8}	UAC,HDL	**2.2X10^{-18}**, 0.147	Bone mineral density	23	*Intergenic*
						UAC	2	*Intergenic*
8	19861214	rs301	6.31x10^{-9}	HDL, ***TG,*** CHE	**4.3X10^{-17}, *4.4X10^{-13}*,** 0.71	HDL	4	*LPL*
8	19863816	rs327	8.72x10^{-9}	HDL, ***TG***, CHE	**9.1X10^{-13}**, ***1.7X10^{-13}***, 0.006	HDL	4	*LPL*
8	19864004	rs328	1.01X10^{-11}	HDL,CRP, TG,INS	**9.6X10^{-13}**, 0.210, **1.6X10^{-15}**, 0.104	TG	14, 24, 25	*LPL*
						HDL	5, 26	*LPL*
8	19864713	rs12679834	1.04x10^{-10}	HDL, ***TG***	**3.2X10^{-12}, *4.0X10^{-15}***	HDL	4	*LPL*
8	19868772	rs13702	6.40 x10^{-09}	HDL, TG, CHE	**2.6X10^{-17},** 3.1X10^{-13}, 0.006	HDL	4	*LPL*
						TG	25	*LPL*

8	19875201	rs10096633	2.92X10^{-13}	HDL,CRP, TG,CHE	***4.8X10^{-12}***, 0.009, **5.0X10^{-15}**, 0.070	TG	[7, 27]	*LPL*
						TC	[9]	*LPL*
8	19876926	rs17482753	1.31X10^{-11}	HDL,CRP, ***TG***,INS	**1.5X10^{-12}**, 0.20, ***3.0X10^{-15}*,** 0.097	HDL	[28]	*LPL*
						HDL	[11]	*LPL*
8	19888502	rs12678919	1.30X10^{-11}	HDL,CRP, TG,INS, CHE	**7.3X10^{-12}**, 0.112, **5.4X10^{-14}**, 0.056, 0.060	TG, HDL	[5, 6]	*LPL*
8	19891970	rs10503669	5.23X10^{-11}	HDL,CRP, TG,INS	**5.10X10^{-12}**, 0.18, **9.10X10^{-15}**, 0.111	HDL	[4, 11, 29]	*LPL*
						TG	[29]	*LPL*
8	19892360	rs17410962	2.80X10^{-13}	HDL,CRP, ***TG***, CHE	**5.1X10^{-12}**, **0.008,** ***5.1X10^{-15}***, 0.069	HDL	[4, 11]	*LPL*
8	19896325	rs17489268	6.35X10^{-9}	HDL,***TG***, CHE	**3.0 X10^{-18}** , ***5.2X10^{-13}*** , 0.023	HDL	[4, 11]	*LPL*

8	19896590	rs17411031	6.35X10^{-9}	HDL,***TG***, CHE	**3.0 X10^{-18}** , ***5.2X10^{-13}*** , 0.023	HDL	[4, 11]	*LPL*
8	19896866	rs4922117	6.35X10^{-9}	HDL,***TG***, CHE	**3.0 X10^{-18}** , ***5.2X10^{-13}*** , 0.023	HDL	[11]	*LPL*
8	19896798	rs17489282	6.35X10^{-9}	HDL,***TG***, GLU,CHE	**3.0X10^{-18}**, ***5.2X10^{-13}***, 0.93 ,0.023	HDL	[4, 11]	*LPL*
8	19899552	rs17411126	6.35X10^{-9}	HDL,***TG***, CHE	**3.0 X10^{-18}**, ***5.2X10^{-13}*** , 0.023	HDL	[4, 11]	*LPL*
8	19909455	rs2083637	1.02X10^{-8}	HDL,***TG***, CHE	**4.8X10^{-18}**, ***9.3X10^{-13}***, 0.027	HDL	[7]	*LPL*
						Waist circumference and related phenotypes	[30]	*LPL*
8	19911725	rs11986942	6.35X10^{-9}	HDL,***TG***, CHE	**1.3 X10^{-16}**, ***6.5 X10^{-13}*** , 0.015	HDL	[4, 11]	*LPL*

8	19912570	rs1837842	61l02X10^{-8}	HDL,***TG***,	**4.8 X10^{-18}** , ***9.3X10^{-13}*** ,	HDL	11	*LPL*
				CHE	0.027			
8	19913956	rs1919484	1.02X10^{-8}	HDL,***TG***,	**4.8X10^{-18}** , ***9.3X10^{-13}*** ,	HDL	4, 11	*LPL*
				CHE	0.027			
11	116154127	rs964184	1.92X10^{-17}	HDL,LDL,	1.7X10^{-6}, 0.049,	HDL	5, 6	*APOA1*
				TG	***7.2X10^{-40}***			
						TC,LDL	6	*APOA1*
						TG	5, 31	*APOA1*
11	116157633	rs6589566	3.351X10^{-9}	HDL,***TG***	0.015, ***2.9X10^{-21}***	LDL	13	*APOA1*,
								APOC3,
								APOA5
11	116158506	rs2075290	3.35X10^{-9}	HDL,***TG***,	0.015, ***2.9X10^{-21}*** ,	APOB	29	*ZNF259*
				CHE	0.035			
11	116165896	rs2266788	3.34X10^{-9}	HDL,***TG***,	0.013, ***2.9X10^{-21}*** ,	APOB	29	*APOA5*
				CHE	0.032			

12	119873345	rs2650000	$9.41X10^{-10}$	LDL,***GGT***, CHE,AST	$2.7X10^{-5}$, ***$3.7X10^{-13}$***, 0.046, 0.24	LDL	[5]	*HNF1A*
						CRP	[9]	*HNF1A*
12	119901033	rs1169288	$3.08X10^{-11}$	LDL,FERR, ***GGT***	$1.6X10^{-5}$, 0.29, ***$1.4X10^{-15}$***	LDL,TC	[6]	*HNF1A*
12	119905190	rs1183910	$1.18X10^{-11}$	LDL,FERR, ***GGT***, TG	$1.3X10^{-5}$, 0.26, ***$8.0X10^{-16}$,*** 0.122	CRP	[32, 33]	*HNF1A*
12	119909244	rs7310409	$2.44X10^{-8}$	CRP, ***GGT***, TG	0.0002, ***$2.5X10^{-13}$***, 0.19	CRP	[34]	*HNF1A*
12	119919970	rs2259816	$2.42X10^{-10}$	LDL,CRP, ***GGT,***TG	$4.5X10^{-5}$, $1.1X10^{-4}$, ***$6.8X10^{-14}$***, 0.31	CAD risk	[35]	*HNF1A, C12orf43*
12	119923816	rs1169310	$2.42X10^{-10}$	LDL,CRP, ***GGT***,TG	$4.5X10^{-5}$, $1.1X10^{-4}$, ***$6.8X10^{-14}$***, 0.31	CRP	[36]	*HNF1A*
						TC,LDL	[6]	*HNF1A*
12	119927053	rs1169313	$2.42X10^{-10}$	LDL, CRP, GGT, TG	$4.5X10^{-05}$, $1.1X10^{-4}$, **$6.8X10^{-14}$**, 0.31	GGT	[37]	*HNF1A,, C12orf43*

15	56461987	rs4775041	$4.65X10^{-8}$	HDL,FERR, TG,INS	$\mathbf{1.8X10^{-8}}$, 0.072, 0.001, 0.610	Serum metabolites	[38]	*LIPC*
						TG, HDL	[29]	*LIPC*
						APOA1	[29]	*LIPC*
15	56465804	rs10468017	$2.75X10^{-11}$	HDL,TG	$\mathbf{2.8X10^{-12}}$, 0.002	HDL	[5]	*LIPC*
15	56470658	rs1532085	$1.85X10^{-9}$	HDL,TG	$\mathbf{2.4X10^{-11}}$, $4.9X10^{-5}$	HDL	[7, 9]	*LIPC*
						TC	[6, 7]	*LIPC*
						TG	[6]	*LIPC*
16	55542640	rs9989419	$2.24X10^{-18}$	HDL,LDL	$\mathbf{2.9X10^{-9}}$, 0.027	HDL	[4, 11, 28, 29]	*CETP*
						APOA1	[4, 29]	*CETP*
16	55545545	rs173539	$1.69X10^{-40}$	HDL,LDL	$\mathbf{3.3X10^{-65}}$, 0.001	HDL	[5]	*CETP*
16	55550712	rs12708967	$3.16X10^{-18}$	HDL,LDL	$\mathbf{1.9X10^{-48}}$, 0.101	HDL, APOA1	[4]	*CETP*

16	55550825	rs3764261	$1.69X10^{-40}$	HDL,LDL	**$3.3X10^{-65}$**, 0.001	Waist circumference and related phenotypes	[30]	*CETP*
						LDL	[6, 39]	*CETP*
						HDL	[4, 6, 9, 16, 29]	*CETP*
						TC,TG	[6]	*CETP*
						APOA1	[4]	*CETP*
16	55552737	rs1800775	$6.72X10^{-32}$	HDL,LDL	**$1.6X10^{-47}$**, 0.0009	HDL	[4, 5, 11, 26, 34]	*CETP*
16	55553712	rs711752	$2.96X10^{-34}$	HDL,LDL	**$2.3X10^{-51}$**, 0.0006	HDL, APOA1	[4]	*CETP*
						TG	[14]	*CETP*
16	55554734	rs1864163	$2.42X10^{-18}$	HDL,LDL, GGT	**$2.9X10^{-45}$**, 0.30, 0.002	HDL	[29]	*CETP*

						HDL,	[4]	*CETP*
						APOA1		
16	55556759	rs7203984	$2.12X10^{-17}$	HDL,TG	**1.1X10^{-46}**, 0.08	HDL,	[4]	*CETP*
						APOA1		
16	55556829	rs11508026	$9.22X10^{-35}$	HDL,LDL	**3.5X10^{-51}**, 0.0009	HDL,	[4]	*CETP*
						APOA1		
16	55560233	rs9939224	$1.28X10^{-19}$	HDL,LDL	**3.3X10^{-51}**, 0.130	HDL,	[4]	*CETP*
						APOA1		
16	55562980	rs1532624	$8.39X10^{-36}$	HDL,LDL,	**2.2X10^{-53}**, 0.0003,	HDL	[4, 7]	*CETP*
				TG	0.0007			
						Cholesterol	[40]	*CETP*
						APOA1	[4]	*CETP*
16	55563879	rs11076175	$1.28X10^{-19}$	HDL,GGT	**3.3X10^{-51}**, 0.002	HDL,	[4]	*CETP*
						APOA1		
16	55564091	rs7499892	$1.28X10^{-19}$	HDL,GGT	**3.3X10^{-51}**, 0.002	HDL	[4, 19]	*CETP*
						APOA1	[4]	*CETP*

16	55564952	rs289714	1.07X10^{-9}	HDL,GGT	**1.1X10^{-29}**, 0.11	HDL	4	*CETP*
19	49934013	rs1531517	4.32X10^{-11}	***LDL***, GLUC	***1.1X10^{-29}***, 0.65	APOB	4	*APOE-APOC*
19	49939467	rs4803750	9.04X10^{-14}	HDL,LDL, GLUC	0.0027, **1.3X10^{-21},** 0.027	LDL APOB	1	*APOE-APOC*
19	50081014	rs283813	5.60X10^{-9}	***LDL***	***2.8X10^{-14}***	APOB	4	*PVRL2*
19	50087459	rs2075650	5.60X10^{-10}	HDL,LDL, CRP,TG	8.1X10^{-8}, **1.6X10^{-14}**, **4.2X10^{-08}**, 9.6X10^{-7}	TC	7	*TOMM40, APOE*
						Alzheimer's disease	41-43	*APOE, TOMM40*
						CRP	36	*APOE, TOMM40*
						Brain imaging	44	*APOE, TOMM40*
						LDL buoyancy	45	*TOMM40*
						LDL		*TOMM40*

						APOB	4	*TOMM40*
19	50114786	rs4420638	7.495X10**$^{-10}$**	HDL,LDL, TG	**1.5X10^{-8}**, **4.3X10^{-14}**, 3.6X10^{-7}	LDL	5, 6, 12, 29, 46, 47	*APOE, APOC1, APOC4, APOC2*
						Alzheimer's disease (late onset)	48-50	*APOE*
						CRP	32, 33, 51	*APOE, APOC1, APOC2*
						TG	14	*APOE*
						HDL, TC	6	*APOE, APOC1, APOC4*
19	50139061	rs12721109	1.40X10**$^{-11}$**	***LDL***, ALT	***3.7X10^{-31}***, 0.67	APOB	4	*APOC4*

Note: SNPs listed in the above table were obtained from the Genome-wide association study (GWAS) catalog [52] (accessed on 9 Aug 2010). Later publications were also added into the table manually.

Table S4: Summary of borderline significant associations (multivariate p-value of < $9x10^{-5}$ and > $5x10^{-8}$)

**CHR**	**BP**	**SNP**	**Closest Gene**	**Minor/ Major Allele**	**MAF**	**N**	**multivariate P-value**	**Associated Trait(s)**	**Univariate analysis**			
									**N**	**Effect**	**SE**	**univariate p**
2	27584444	rs1260326[1]	GCKR	T/C	0.400	4883	$1.9 x10^{-6}$	**TRIG**	11578	0.082	0.015	**$2.0 x10^{-8}$**
								UA	11353	0.056	0.014	$7.0 x10^{-5}$
								FERR	11331	–0.011	0.014	0.440
								ALT	11517	–0.030	0.014	0.037
4	89264355	rs2199936[2]	ABCG2	A/G	0.116	4884	$9.2 x10^{-7}$	**UA**	11350	0.188	0.022	**$1.6 x10^{-17}$**
								CRP	8918	0.025	0.024	0.300
								FERR	11328	–0.031	0.022	0.150
								TRIG	11575	0.034	0.023	0.132
								CHE	9168	–0.051	0.023	0.030
6	25884928	rs11754288[2]	SLC17A4	A/G	0.428	4884	$1.1 x10^{-5}$	**UA**	11354	–0.084	0.014	**$4.4 x10^{-9}$**
								HDL	11549		0.014	0.680

								LDL	11251	–0.006	0.015	0.094
								INS	2560	0.024	0.034	0.112
										0.054		
6	26201120	rs1800562 [3]	HFE	A/G	0.075	4884	5.8 $x10^{-7}$	**FERR**	11332	0.177	0.026	**4.7 $x10^{-12}$**
								HDL	11549	0.050	0.026	0.056
								LDL	11251	–0.071	0.027	0.008
								TG	11251	–0.017	0.027	0.052
11	116580776	rs508487	PCSK7	T/C	0.040	4876	2.7 $x10^{-5}$	**TRIG**	11557	0.235	0.037	**2.2 $x10^{-10}$**
								LDL	11230	0.110	0.037	0.003
								GGT	11494	0.051	0.036	0.150
19	11063306	rs6511720[1]	LDLR	T/G	0.114	4877	7.1 $x10^{-5}$	**LDL**	11238	–0.148	0.023	**5.0 $x10^{-11}$**
								AST	11503	–0.027	0.022	0.220

Additional References

1. Chasman DI, Pare G, Mora S, Hopewell JC, Peloso G, Clarke R, Cupples LA, Hamsten A, Kathiresan S, Malarstig A, Ordovas JM, Ripatti S, Parker AN, Miletich JP, Ridker PM. Forty-three loci associated with plasma lipoprotein size, concentration, and cholesterol content in genome-wide analysis. *PLoS Genet*. 2009;5: e1000730.

2. Kolz M, Johnson T, Sanna S, Teumer A, Vitart V, Perola M, Mangino M, Albrecht E, Wallace C, Farrall M, Johansson A, Nyholt DR, Aulchenko Y, Beckmann JS, Bergmann S, Bochud M, Brown M, Campbell H, Connell J, Dominiczak A, Homuth G, Lamina C, McCarthy MI, Meitinger T, Mooser V, Munroe P, Nauck M, Peden J, Prokisch H, Salo P, Salomaa V, Samani NJ, Schlessinger D, Uda M, Volker U, Waeber G, Waterworth D, Wang-Sattler R, Wright AF, Adamski J, Whitfield JB, Gyllensten U, Wilson JF, Rudan I, Pramstaller P, Watkins H, Doering A, Wichmann HE, Spector TD, Peltonen L, Volzke H, Nagaraja R, Vollenweider P, Caulfield M, Illig T, Gieger C. Meta-analysis of 28,141 individuals identifies common variants within five new loci that influence uric acid concentrations. *PLoS Genet*. 2009;5: e1000504.

3. Benyamin B, McRae AF, Zhu G, Gordon S, Henders AK, Palotie A, Peltonen L, Martin NG, Montgomery GW, Whitfield JB, Visscher PM. Variants in tf and hfe explain approximately 40% of genetic variation in serum-transferrin levels. *Am J Hum Genet*. 2009;84: 60-65.

4. Talmud PJ, Drenos F, Shah S, Shah T, Palmen J, Verzilli C, Gaunt TR, Pallas J, Lovering R, Li K, Casas JP, Sofat R, Kumari M, Rodriguez S, Johnson T, Newhouse SJ, Dominiczak A, Samani NJ, Caulfield M, Sever P, Stanton A, Shields DC, Padmanabhan S, Melander O, Hastie C, Delles C, Ebrahim S, Marmot MG, Smith GD, Lawlor DA, Munroe PB, Day IN, Kivimaki M, Whittaker J, Humphries SE, Hingorani AD. Gene-centric association signals for lipids and apolipoproteins identified via the humancvd beadchip. *Am J Hum Genet*. 2009;85: 628-642.

5. Kathiresan S, Melander O, Guiducci C, Surti A, Burtt NP, Rieder MJ, Cooper GM, Roos C, Voight BF, Havulinna AS, Wahlstrand B, Hedner T, Corella D, Tai ES, Ordovas JM, Berglund G, Vartiainen E, Jousilahti P, Hedblad B, Taskinen MR, Newton-Cheh C, Salomaa V, Peltonen L, Groop L, Altshuler DM, Orho-Melander M. Six new loci associated with blood low-density lipoprotein cholesterol, high-density lipoprotein cholesterol or triglycerides in humans. *Nat Genet*. 2008;40: 189-197.

6. Teslovich TM, Musunuru K, Smith AV, Edmondson AC, Stylianou IM, Koseki M, Pirruccello JP, Ripatti S, Chasman DI, Willer CJ, Johansen CT, Fouchier SW, Isaacs A, Peloso GM, Barbalic M, Ricketts SL, Bis JC, Aulchenko YS, Thorleifsson G, Feitosa MF, Chambers J, Orho-Melander M, Melander O, Johnson T, Li X, Guo X, Li M, Shin Cho Y, Jin Go M, Jin Kim Y, Lee JY, Park T, Kim K, Sim X, Twee-Hee Ong R, Croteau-Chonka DC, Lange LA, Smith JD, Song K, Hua Zhao J, Yuan X, Luan J, Lamina C, Ziegler A, Zhang W, Zee RY, Wright AF, Witteman JC, Wilson JF, Willemsen G, Wichmann HE, Whitfield JB, Waterworth DM, Wareham NJ, Waeber G, Vollenweider P, Voight BF, Vitart V, Uitterlinden AG, Uda M, Tuomilehto J, Thompson JR, Tanaka T, Surakka I, Stringham HM, Spector TD, Soranzo N, Smit JH, Sinisalo J, Silander K, Sijbrands EJ, Scuteri A, Scott J, Schlessinger D, Sanna S,

Salomaa V, Saharinen J, Sabatti C, Ruokonen A, Rudan I, Rose LM, Roberts R, Rieder M, Psaty BM, Pramstaller PP, Pichler I, Perola M, Penninx BW, Pedersen NL, Pattaro C, Parker AN, Pare G, Oostra BA, O'Donnell CJ, Nieminen MS, Nickerson DA, Montgomery GW, Meitinger T, McPherson R, McCarthy MI, McArdle W, Masson D, Martin NG, Marroni F, Mangino M, Magnusson PK, Lucas G, Luben R, Loos RJ, Lokki ML, Lettre G, Langenberg C, Launer LJ, Lakatta EG, Laaksonen R, Kyvik KO, Kronenberg F, Konig IR, Khaw KT, Kaprio J, Kaplan LM, Johansson A, Jarvelin MR, Cecile JWJA, Ingelsson E, Igl W, Kees Hovingh G, Hottenga JJ, Hofman A, Hicks AA, Hengstenberg C, Heid IM, Hayward C, Havulinna AS, Hastie ND, Harris TB, Haritunians T, Hall AS, Gyllensten U, Guiducci C, Groop LC, Gonzalez E, Gieger C, Freimer NB, Ferrucci L, Erdmann J, Elliott P, Ejebe KG, Doring A, Dominiczak AF, Demissie S, Deloukas P, de Geus EJ, de Faire U, Crawford G, Collins FS, Chen YD, Caulfield MJ, Campbell H, Burtt NP, Bonnycastle LL, Boomsma DI, Boekholdt SM, Bergman RN, Barroso I, Bandinelli S, Ballantyne CM, Assimes TL, Quertermous T, Altshuler D, Seielstad M, Wong TY, Tai ES, Feranil AB, Kuzawa CW, Adair LS, Taylor HA, Jr., Borecki IB, Gabriel SB, Wilson JG, Holm H, Thorsteinsdottir U, Gudnason V, Krauss RM, Mohlke KL, Ordovas JM, Munroe PB, Kooner JS, Tall AR, Hegele RA, Kastelein JJ, Schadt EE, Rotter JI, Boerwinkle E, Strachan DP, Mooser V, Stefansson K, Reilly MP, Samani NJ, Schunkert H, Cupples LA, Sandhu MS, Ridker PM, Rader DJ, van Duijn CM, Peltonen L, Abecasis GR, Boehnke M, Kathiresan S. Biological, clinical and population relevance of 95 loci for blood lipids. *Nature*. 2010;466: 707-713.

7. Aulchenko YS, Ripatti S, Lindqvist I, Boomsma D, Heid IM, Pramstaller PP, Penninx BW, Janssens AC, Wilson JF, Spector T, Martin NG, Pedersen NL, Kyvik KO,

Kaprio J, Hofman A, Freimer NB, Jarvelin MR, Gyllensten U, Campbell H, Rudan I, Johansson A, Marroni F, Hayward C, Vitart V, Jonasson I, Pattaro C, Wright A, Hastie N, Pichler I, Hicks AA, Falchi M, Willemsen G, Hottenga JJ, de Geus EJ, Montgomery GW, Whitfield J, Magnusson P, Saharinen J, Perola M, Silander K, Isaacs A, Sijbrands EJ, Uitterlinden AG, Witteman JC, Oostra BA, Elliott P, Ruokonen A, Sabatti C, Gieger C, Meitinger T, Kronenberg F, Doring A, Wichmann HE, Smit JH, McCarthy MI, van Duijn CM, Peltonen L. Loci influencing lipid levels and coronary heart disease risk in 16 european population cohorts. *Nat Genet.* 2009;41: 47-55.

8. Kathiresan S, Voight BF, Purcell S, Musunuru K, Ardissino D, Mannucci PM, Anand S, Engert JC, Samani NJ, Schunkert H, Erdmann J, Reilly MP, Rader DJ, Morgan T, Spertus JA, Stoll M, Girelli D, McKeown PP, Patterson CC, Siscovick DS, O'Donnell CJ, Elosua R, Peltonen L, Salomaa V, Schwartz SM, Melander O, Altshuler D, Merlini PA, Berzuini C, Bernardinelli L, Peyvandi F, Tubaro M, Celli P, Ferrario M, Fetiveau R, Marziliano N, Casari G, Galli M, Ribichini F, Rossi M, Bernardi F, Zonzin P, Piazza A, Yee J, Friedlander Y, Marrugat J, Lucas G, Subirana I, Sala J, Ramos R, Meigs JB, Williams G, Nathan DM, MacRae CA, Havulinna AS, Berglund G, Hirschhorn JN, Asselta R, Duga S, Spreafico M, Daly MJ, Nemesh J, Korn JM, McCarroll SA, Surti A, Guiducci C, Gianniny L, Mirel D, Parkin M, Burtt N, Gabriel SB, Thompson JR, Braund PS, Wright BJ, Balmforth AJ, Ball SG, Hall AS, Linsel-Nitschke P, Lieb W, Ziegler A, Konig I, Hengstenberg C, Fischer M, Stark K, Grosshennig A, Preuss M, Wichmann HE, Schreiber S, Ouwehand W, Deloukas P, Scholz M, Cambien F, Li M, Chen Z, Wilensky R, Matthai W, Qasim A, Hakonarson HH, Devaney J, Burnett MS, Pichard AD, Kent KM, Satler L, Lindsay JM, Waksman

R, Epstein SE, Scheffold T, Berger K, Huge A, Martinelli N, Olivieri O, Corrocher R, McKeown P, Erdmann E, Konig IR, Holm H, Thorleifsson G, Thorsteinsdottir U, Stefansson K, Do R, Xie C, Siscovick D. Genome-wide association of early-onset myocardial infarction with single nucleotide polymorphisms and copy number variants. *Nat Genet*. 2009;41: 334-341.

9. Sabatti C, Service SK, Hartikainen AL, Pouta A, Ripatti S, Brodsky J, Jones CG, Zaitlen NA, Varilo T, Kaakinen M, Sovio U, Ruokonen A, Laitinen J, Jakkula E, Coin L, Hoggart C, Collins A, Turunen H, Gabriel S, Elliot P, McCarthy MI, Daly MJ, Jarvelin MR, Freimer NB, Peltonen L. Genome-wide association analysis of metabolic traits in a birth cohort from a founder population. *Nat Genet*. 2009;41: 35-46.

10. Samani NJ, Erdmann J, Hall AS, Hengstenberg C, Mangino M, Mayer B, Dixon RJ, Meitinger T, Braund P, Wichmann HE, Barrett JH, Konig IR, Stevens SE, Szymczak S, Tregouet DA, Iles MM, Pahlke F, Pollard H, Lieb W, Cambien F, Fischer M, Ouwehand W, Blankenberg S, Balmforth AJ, Baessler A, Ball SG, Strom TM, Braenne I, Gieger C, Deloukas P, Tobin MD, Ziegler A, Thompson JR, Schunkert H. Genomewide association analysis of coronary artery disease. *N Engl J Med*. 2007;357: 443-453.

11. Ma L, Yang J, Runesha HB, Tanaka T, Ferrucci L, Bandinelli S, Da Y. Genome-wide association analysis of total cholesterol and high-density lipoprotein cholesterol levels using the framingham heart study data. *BMC Med Genet*. 2010;11: 55.

12. Sandhu MS, Waterworth DM, Debenham SL, Wheeler E, Papadakis K, Zhao JH, Song K, Yuan X, Johnson T, Ashford S, Inouye M, Luben R, Sims M, Hadley D, McArdle W, Barter P, Kesaniemi YA, Mahley RW, McPherson R, Grundy SM, Bingham SA, Khaw KT, Loos RJ, Waeber G, Barroso I, Strachan DP, Deloukas P, Vollenweider P, Wareham NJ, Mooser V. Ldl-cholesterol concentrations: A genome-wide association study. *Lancet*. 2008;371: 483-491.

13. Wallace C, Newhouse SJ, Braund P, Zhang F, Tobin M, Falchi M, Ahmadi K, Dobson RJ, Marcano AC, Hajat C, Burton P, Deloukas P, Brown M, Connell JM, Dominiczak A, Lathrop GM, Webster J, Farrall M, Spector T, Samani NJ, Caulfield MJ, Munroe PB. Genome-wide association study identifies genes for biomarkers of cardiovascular disease: Serum urate and dyslipidemia. *Am J Hum Genet*. 2008;82: 139-149.

14. Saxena R, Voight BF, Lyssenko V, Burtt NP, de Bakker PI, Chen H, Roix JJ, Kathiresan S, Hirschhorn JN, Daly MJ, Hughes TE, Groop L, Altshuler D, Almgren P, Florez JC, Meyer J, Ardlie K, Bengtsson Bostrom K, Isomaa B, Lettre G, Lindblad U, Lyon HN, Melander O, Newton-Cheh C, Nilsson P, Orho-Melander M, Rastam L, Speliotes EK, Taskinen MR, Tuomi T, Guiducci C, Berglund A, Carlson J, Gianniny L, Hackett R, Hall L, Holmkvist J, Laurila E, Sjogren M, Sterner M, Surti A, Svensson M, Tewhey R, Blumenstiel B, Parkin M, Defelice M, Barry R, Brodeur W, Camarata J, Chia N, Fava M, Gibbons J, Handsaker B, Healy C, Nguyen K, Gates C, Sougnez C, Gage D, Nizzari M, Gabriel SB, Chirn GW, Ma Q, Parikh H, Richardson D, Ricke D, Purcell S. Genome-wide association analysis identifies loci for type 2 diabetes and triglyceride levels. *Science*. 2007;316: 1331-1336.

15. Benyamin B, Middelberg RP, Lind PA, Valle AM, Gordon S, Medland SE, Henders AK, Wray NR, Heath AC, Madden PAF, Visscher PM, O'Connor D T, Montgomery GW, Martin NG, Whitfield JB. Genomewide association study of butyrylcholinesterase activity and its association with metabolic and cardiovascular risk factors. . (Submitted)

16. Kamatani Y, Matsuda K, Okada Y, Kubo M, Hosono N, Daigo Y, Nakamura Y, Kamatani N. Genome-wide association study of hematological and biochemical traits in a japanese population. *Nat Genet*. 2010;42: 210-215.

17. Dehghan A, Kottgen A, Yang Q, Hwang SJ, Kao WL, Rivadeneira F, Boerwinkle E, Levy D, Hofman A, Astor BC, Benjamin EJ, van Duijn CM, Witteman JC, Coresh J, Fox CS. Association of three genetic loci with uric acid concentration and risk of gout: A genome-wide association study. *Lancet*. 2008;372: 1953-1961.

18. McArdle PF, Parsa A, Chang YP, Weir MR, O'Connell JR, Mitchell BD, Shuldiner AR. Association of a common nonsynonymous variant in glut9 with serum uric acid levels in old order amish. *Arthritis Rheum*. 2008;58: 2874-2881.

19. Zemunik T, Boban M, Lauc G, Jankovic S, Rotim K, Vatavuk Z, Bencic G, Dogas Z, Boraska V, Torlak V, Susac J, Zobic I, Rudan D, Pulanic D, Modun D, Mudnic I, Gunjaca G, Budimir D, Hayward C, Vitart V, Wright AF, Campbell H, Rudan I. Genome-wide association study of biochemical traits in korcula island, croatia. *Croat Med J*. 2009;50: 23-33.

20. Vitart V, Rudan I, Hayward C, Gray NK, Floyd J, Palmer CN, Knott SA, Kolcic I, Polasek O, Graessler J, Wilson JF, Marinaki A, Riches PL, Shu X, Janicijevic B, Smolej-Narancic N, Gorgoni B, Morgan J, Campbell S, Biloglav Z, Barac-Lauc L, Pericic M, Klaric IM, Zgaga L, Skaric-Juric T, Wild SH, Richardson WA, Hohenstein P, Kimber CH, Tenesa A, Donnelly LA, Fairbanks LD, Aringer M, McKeigue PM, Ralston SH, Morris AD, Rudan P, Hastie ND, Campbell H, Wright AF. Slc2a9 is a newly identified urate transporter influencing serum urate concentration, urate excretion and gout. *Nat Genet*. 2008;40: 437-442.

21. Li S, Sanna S, Maschio A, Busonero F, Usala G, Mulas A, Lai S, Dei M, Orru M, Albai G, Bandinelli S, Schlessinger D, Lakatta E, Scuteri A, Najjar SS, Guralnik J, Naitza S, Crisponi L, Cao A, Abecasis G, Ferrucci L, Uda M, Chen WM, Nagaraja R. The glut9 gene is associated with serum uric acid levels in sardinia and chianti cohorts. *PLoS Genet*. 2007;3: e194.

22. Doring A, Gieger C, Mehta D, Gohlke H, Prokisch H, Coassin S, Fischer G, Henke K, Klopp N, Kronenberg F, Paulweber B, Pfeufer A, Rosskopf D, Volzke H, Illig T, Meitinger T, Wichmann HE, Meisinger C. Slc2a9 influences uric acid concentrations with pronounced sex-specific effects. *Nat Genet*. 2008;40: 430-436.

23. Kiel DP, Demissie S, Dupuis J, Lunetta KL, Murabito JM, Karasik D. Genome-wide association with bone mass and geometry in the framingham heart study. *BMC Med Genet*. 2007;8 Suppl 1: S14.

24. Kathiresan S, Manning AK, Demissie S, D'Agostino RB, Surti A, Guiducci C, Gianniny L, Burtt NP, Melander O, Orho-Melander M, Arnett DK, Peloso GM,

Ordovas JM, Cupples LA. A genome-wide association study for blood lipid phenotypes in the framingham heart study. *BMC Med Genet*. 2007;8 Suppl 1: S17.

25. Lanktree MB, Anand SS, Yusuf S, Hegele RA. Replication of genetic associations with plasma lipoprotein traits in a multiethnic sample. *J Lipid Res*. 2009;50: 1487-1496.

26. Keebler ME, Sanders CL, Surti A, Guiducci C, Burtt NP, Kathiresan S. Association of blood lipids with common DNA sequence variants at 19 genetic loci in the multiethnic united states national health and nutrition examination survey iii. *Circ Cardiovasc Genet*. 2009;2: 238-243.

27. Deo RC, Reich D, Tandon A, Akylbekova E, Patterson N, Waliszewska A, Kathiresan S, Sarpong D, Taylor HA, Jr., Wilson JG. Genetic differences between the determinants of lipid profile phenotypes in african and european americans: The jackson heart study. *PLoS Genet*. 2009;5: e1000342.

28. Heid IM, Boes E, Muller M, Kollerits B, Lamina C, Coassin S, Gieger C, Doring A, Klopp N, Frikke-Schmidt R, Tybjaerg-Hansen A, Brandstatter A, Luchner A, Meitinger T, Wichmann HE, Kronenberg F. Genome-wide association analysis of high-density lipoprotein cholesterol in the population-based kora study sheds new light on intergenic regions. *Circ Cardiovasc Genet*. 2008;1: 10-20.

29. Willer CJ, Sanna S, Jackson AU, Scuteri A, Bonnycastle LL, Clarke R, Heath SC, Timpson NJ, Najjar SS, Stringham HM, Strait J, Duren WL, Maschio A, Busonero F, Mulas A, Albai G, Swift AJ, Morken MA, Narisu N, Bennett D, Parish S, Shen H,

Galan P, Meneton P, Hercberg S, Zelenika D, Chen WM, Li Y, Scott LJ, Scheet PA, Sundvall J, Watanabe RM, Nagaraja R, Ebrahim S, Lawlor DA, Ben-Shlomo Y, Davey-Smith G, Shuldiner AR, Collins R, Bergman RN, Uda M, Tuomilehto J, Cao A, Collins FS, Lakatta E, Lathrop GM, Boehnke M, Schlessinger D, Mohlke KL, Abecasis GR. Newly identified loci that influence lipid concentrations and risk of coronary artery disease. *Nat Genet*. 2008;40: 161-169.

30. Chambers JC, Elliott P, Zabaneh D, Zhang W, Li Y, Froguel P, Balding D, Scott J, Kooner JS. Common genetic variation near mc4r is associated with waist circumference and insulin resistance. *Nat Genet*. 2008;40: 716-718.

31. Weissglas-Volkov D, Aguilar-Salinas CA, Sinsheimer JS, Riba L, Huertas-Vazquez A, Ordonez-Sanchez ML, Rodriguez-Guillen R, Cantor RM, Tusie-Luna T, Pajukanta P. Investigation of variants identified in caucasian genome-wide association studies for plasma high-density lipoprotein cholesterol and triglycerides levels in mexican dyslipidemic study samples. *Circ Cardiovasc Genet*. 2010;3: 31-38.

32. Elliott P, Chambers JC, Zhang W, Clarke R, Hopewell JC, Peden JF, Erdmann J, Braund P, Engert JC, Bennett D, Coin L, Ashby D, Tzoulaki I, Brown IJ, Mt-Isa S, McCarthy MI, Peltonen L, Freimer NB, Farrall M, Ruokonen A, Hamsten A, Lim N, Froguel P, Waterworth DM, Vollenweider P, Waeber G, Jarvelin MR, Mooser V, Scott J, Hall AS, Schunkert H, Anand SS, Collins R, Samani NJ, Watkins H, Kooner JS. Genetic loci associated with c-reactive protein levels and risk of coronary heart disease. *Jama*. 2009;302: 37-48.

33. Dehghan A, Dupuis J, Barbalic M, Bis JC, Eiriksdottir G, Lu C, Pellikka N, Wallaschofski H, Kettunen J, Henneman P, Baumert J, Strachan DP, Fuchsberger C, Vitart V, Wilson JF, Pare G, Naitza S, Rudock ME, Surakka I, de Geus EJ, Alizadeh BZ, Guralnik J, Shuldiner A, Tanaka T, Zee RY, Schnabel RB, Nambi V, Kavousi M, Ripatti S, Nauck M, Smith NL, Smith AV, Sundvall J, Scheet P, Liu Y, Ruokonen A, Rose LM, Larson MG, Hoogeveen RC, Freimer NB, Teumer A, Tracy RP, Launer LJ, Buring JE, Yamamoto JF, Folsom AR, Sijbrands EJ, Pankow J, Elliott P, Keaney JF, Sun W, Sarin AP, Fontes JD, Badola S, Astor BC, Hofman A, Pouta A, Werdan K, Greiser KH, Kuss O, Meyer Zu Schwabedissen HE, Thiery J, Jamshidi Y, Nolte IM, Soranzo N, Spector TD, Volzke H, Parker AN, Aspelund T, Bates D, Young L, Tsui K, Siscovick DS, Guo X, Rotter JI, Uda M, Schlessinger D, Rudan I, Hicks AA, Penninx BW, Thorand B, Gieger C, Coresh J, Willemsen G, Harris TB, Uitterlinden AG, Jarvelin MR, Rice K, Radke D, Salomaa V, Willems van Dijk K, Boerwinkle E, Vasan RS, Ferrucci L, Gibson QD, Bandinelli S, Snieder H, Boomsma DI, Xiao X, Campbell H, Hayward C, Pramstaller PP, van Duijn CM, Peltonen L, Psaty BM, Gudnason V, Ridker PM, Homuth G, Koenig W, Ballantyne CM, Witteman JC, Benjamin EJ, Perola M, Chasman DI. Meta-analysis of genome-wide association studies in >80 000 subjects identifies multiple loci for c-reactive protein levels. *Circulation*. 2011: 731-738.

34. Ridker PM, Pare G, Parker A, Zee RY, Danik JS, Buring JE, Kwiatkowski D, Cook NR, Miletich JP, Chasman DI. Loci related to metabolic-syndrome pathways including lepr,hnf1a, il6r, and gckr associate with plasma c-reactive protein: The women's genome health study. *Am J Hum Genet*. 2008;82: 1185-1192.

35. Erdmann J, Grosshennig A, Braund PS, Konig IR, Hengstenberg C, Hall AS, Linsel-Nitschke P, Kathiresan S, Wright B, Tregouet DA, Cambien F, Bruse P, Aherrahrou Z, Wagner AK, Stark K, Schwartz SM, Salomaa V, Elosua R, Melander O, Voight BF, O'Donnell CJ, Peltonen L, Siscovick DS, Altshuler D, Merlini PA, Peyvandi F, Bernardinelli L, Ardissino D, Schillert A, Blankenberg S, Zeller T, Wild P, Schwarz DF, Tiret L, Perret C, Schreiber S, El Mokhtari NE, Schafer A, Marz W, Renner W, Bugert P, Kluter H, Schrezenmeir J, Rubin D, Ball SG, Balmforth AJ, Wichmann HE, Meitinger T, Fischer M, Meisinger C, Baumert J, Peters A, Ouwehand WH, Deloukas P, Thompson JR, Ziegler A, Samani NJ, Schunkert H. New susceptibility locus for coronary artery disease on chromosome 3q22.3. *Nat Genet*. 2009;41: 280-282.

36. Reiner AP, Barber MJ, Guan Y, Ridker PM, Lange LA, Chasman DI, Walston JD, Cooper GM, Jenny NS, Rieder MJ, Durda JP, Smith JD, Novembre J, Tracy RP, Rotter JI, Stephens M, Nickerson DA, Krauss RM. Polymorphisms of the hnf1a gene encoding hepatocyte nuclear factor-1 alpha are associated with c-reactive protein. *Am J Hum Genet*. 2008;82: 1193-1201.

37. Yuan X, Waterworth D, Perry JR, Lim N, Song K, Chambers JC, Zhang W, Vollenweider P, Stirnadel H, Johnson T, Bergmann S, Beckmann ND, Li Y, Ferrucci L, Melzer D, Hernandez D, Singleton A, Scott J, Elliott P, Waeber G, Cardon L, Frayling TM, Kooner JS, Mooser V. Population-based genome-wide association studies reveal six loci influencing plasma levels of liver enzymes. *Am J Hum Genet*. 2008;83: 520-528.

38. Gieger C, Geistlinger L, Altmaier E, Hrabe de Angelis M, Kronenberg F, Meitinger T, Mewes HW, Wichmann HE, Weinberger KM, Adamski J, Illig T, Suhre K.

Genetics meets metabolomics: A genome-wide association study of metabolite profiles in human serum. *PLoS Genet*. 2008;4: e1000282.

39. Hiura Y, Shen CS, Kokubo Y, Okamura T, Morisaki T, Tomoike H, Yoshida T, Sakamoto H, Goto Y, Nonogi H, Iwai N. Identification of genetic markers associated with high-density lipoprotein-cholesterol by genome-wide screening in a japanese population: The suita study. *Circ J*. 2009;73: 1119-1126.

40. Igl W, Johansson A, Wilson JF, Wild SH, Polasek O, Hayward C, Vitart V, Hastie N, Rudan P, Gnewuch C, Schmitz G, Meitinger T, Pramstaller PP, Hicks AA, Oostra BA, van Duijn CM, Rudan I, Wright A, Campbell H, Gyllensten U. Modeling of environmental effects in genome-wide association studies identifies slc2a2 and hp as novel loci influencing serum cholesterol levels. *PLoS Genet*. 2010;6: e1000798.

41. Harold D, Abraham R, Hollingworth P, Sims R, Gerrish A, Hamshere ML, Pahwa JS, Moskvina V, Dowzell K, Williams A, Jones N, Thomas C, Stretton A, Morgan AR, Lovestone S, Powell J, Proitsi P, Lupton MK, Brayne C, Rubinsztein DC, Gill M, Lawlor B, Lynch A, Morgan K, Brown KS, Passmore PA, Craig D, McGuinness B, Todd S, Holmes C, Mann D, Smith AD, Love S, Kehoe PG, Hardy J, Mead S, Fox N, Rossor M, Collinge J, Maier W, Jessen F, Schurmann B, van den Bussche H, Heuser I, Kornhuber J, Wiltfang J, Dichgans M, Frolich L, Hampel H, Hull M, Rujescu D, Goate AM, Kauwe JS, Cruchaga C, Nowotny P, Morris JC, Mayo K, Sleegers K, Bettens K, Engelborghs S, De Deyn PP, Van Broeckhoven C, Livingston G, Bass NJ, Gurling H, McQuillin A, Gwilliam R, Deloukas P, Al-Chalabi A, Shaw CE, Tsolaki M, Singleton AB, Guerreiro R, Muhleisen TW, Nothen MM, Moebus S, Jockel KH, Klopp N, Wichmann HE, Carrasquillo MM, Pankratz VS, Younkin SG, Holmans PA,

O'Donovan M, Owen MJ, Williams J. Genome-wide association study identifies variants at clu and picalm associated with alzheimer's disease. *Nat Genet*. 2009;41: 1088-1093.

42. Heinzen EL, Need AC, Hayden KM, Chiba-Falek O, Roses AD, Strittmatter WJ, Burke JR, Hulette CM, Welsh-Bohmer KA, Goldstein DB. Genome-wide scan of copy number variation in late-onset alzheimer's disease. *J Alzheimers Dis*. 2009

43. Lambert JC, Heath S, Even G, Campion D, Sleegers K, Hiltunen M, Combarros O, Zelenika D, Bullido MJ, Tavernier B, Letenneur L, Bettens K, Berr C, Pasquier F, Fievet N, Barberger-Gateau P, Engelborghs S, De Deyn P, Mateo I, Franck A, Helisalmi S, Porcellini E, Hanon O, de Pancorbo MM, Lendon C, Dufouil C, Jaillard C, Leveillard T, Alvarez V, Bosco P, Mancuso M, Panza F, Nacmias B, Bossu P, Piccardi P, Annoni G, Seripa D, Galimberti D, Hannequin D, Licastro F, Soininen H, Ritchie K, Blanche H, Dartigues JF, Tzourio C, Gut I, Van Broeckhoven C, Alperovitch A, Lathrop M, Amouyel P. Genome-wide association study identifies variants at clu and cr1 associated with alzheimer's disease. *Nat Genet*. 2009;41: 1094-1099.

44. Shen L, Kim S, Risacher SL, Nho K, Swaminathan S, West JD, Foroud T, Pankratz N, Moore JH, Sloan CD, Huentelman MJ, Craig DW, Dechairo BM, Potkin SG, Jack CR, Jr., Weiner MW, Saykin AJ. Whole genome association study of brain-wide imaging phenotypes for identifying quantitative trait loci in mci and ad: A study of the adni cohort. *Neuroimage*. 2010

45. Ronald J, Rajagopalan R, Ranchalis JE, Marshall JK, Hatsukami TS, Heagerty PJ, Jarvik GP. Analysis of recently identified dyslipidemia alleles reveals two loci that contribute to risk for carotid artery disease. *Lipids Health Dis*. 2009;8: 52.

46. Burkhardt R, Kenny EE, Lowe JK, Birkeland A, Josowitz R, Noel M, Salit J, Maller JB, Pe'er I, Daly MJ, Altshuler D, Stoffel M, Friedman JM, Breslow JL. Common snps in hmgcr in micronesians and whites associated with ldl-cholesterol levels affect alternative splicing of exon13. *Arterioscler Thromb Vasc Biol*. 2008;28: 2078-2084.

47. Lowe JK, Maller JB, Pe'er I, Neale BM, Salit J, Kenny EE, Shea JL, Burkhardt R, Smith JG, Ji W, Noel M, Foo JN, Blundell ML, Skilling V, Garcia L, Sullivan ML, Lee HE, Labek A, Ferdowsian H, Auerbach SB, Lifton RP, Newton-Cheh C, Breslow JL, Stoffel M, Daly MJ, Altshuler DM, Friedman JM. Genome-wide association studies in an isolated founder population from the pacific island of kosrae. *PLoS Genet*. 2009;5: e1000365.

48. Coon KD, Myers AJ, Craig DW, Webster JA, Pearson JV, Lince DH, Zismann VL, Beach TG, Leung D, Bryden L, Halperin RF, Marlowe L, Kaleem M, Walker DG, Ravid R, Heward CB, Rogers J, Papassotiropoulos A, Reiman EM, Hardy J, Stephan DA. A high-density whole-genome association study reveals that apoe is the major susceptibility gene for sporadic late-onset alzheimer's disease. *J Clin Psychiatry*. 2007;68: 613-618.

49. Webster JA, Myers AJ, Pearson JV, Craig DW, Hu-Lince D, Coon KD, Zismann VL, Beach T, Leung D, Bryden L, Halperin RF, Marlowe L, Kaleem M, Huentelman MJ, Joshipura K, Walker D, Heward CB, Ravid R, Rogers J, Papassotiropoulos A, Hardy

J, Reiman EM, Stephan DA. Sorl1 as an alzheimer's disease predisposition gene? *Neurodegener Dis*. 2008;5: 60-64.

50. Li H, Wetten S, Li L, St Jean PL, Upmanyu R, Surh L, Hosford D, Barnes MR, Briley JD, Borrie M, Coletta N, Delisle R, Dhalla D, Ehm MG, Feldman HH, Fornazzari L, Gauthier S, Goodgame N, Guzman D, Hammond S, Hollingworth P, Hsiung GY, Johnson J, Kelly DD, Keren R, Kertesz A, King KS, Lovestone S, Loy-English I, Matthews PM, Owen MJ, Plumpton M, Pryse-Phillips W, Prinjha RK, Richardson JC, Saunders A, Slater AJ, St George-Hyslop PH, Stinnett SW, Swartz JE, Taylor RL, Wherrett J, Williams J, Yarnall DP, Gibson RA, Irizarry MC, Middleton LT, Roses AD. Candidate single-nucleotide polymorphisms from a genomewide association study of alzheimer disease. *Arch Neurol*. 2008;65: 45-53.

51. Okada Y, Takahashi A, Ohmiya H, Kumasaka N, Kamatani Y, Hosono N, Tsunoda T, Matsuda K, Tanaka T, Kubo M, Nakamura Y, Yamamoto K, Kamatani N. Genome-wide association study for c-reactive protein levels identified pleiotropic associations in the il6 locus. *Hum Mol Genet*. 2011;20: 1224-1231.

52. Hindorff LA, Junkins HA, Hall PN, Mehta JP, Manolio TA. A catalog of published genome-wide association studies. Available at: www.genome.gov/gwasstudies. Accessed [9 Aug 2010].
